# Antibacterial and Antibiofilm Efficacy of Antimicrobial Photodynamic Therapy Against Intracanal *Enterococcus faecalis*: An *In Vitro* Comparative Study with Traditional Endodontic Irrigation Solutions

**Published:** 2018-07

**Authors:** Maryam Pourhajibagher, Nasim Chiniforush, Sima Shahabi, Morteza Palizvani, Abbas Bahador

**Affiliations:** 1 Researcher, Dental Research Center, Dentistry Research Institute, Tehran University of Medical Sciences, Tehran, Iran; 2 Researcher, Laser Research Center of Dentistry, Dentistry Research Institute, Tehran University of Medical Sciences, Tehran, Iran; 3 Professor, Laser Research Center of Dentistry, Dentistry Research Institute, Tehran University of Medical Sciences, Tehran, Iran; Department of Dental Biomaterials, School of Dentistry, Tehran University of Medical Sciences, Tehran, Iran; 4 Dentist, Private Practice, Tehran, Iran; 5 Associate Professor, Department of Microbiology, School of Medicine, Tehran University of Medical Sciences, Tehran, Iran

**Keywords:** Photochemotherapy, Biofilms, Curcumin, *Enterococcus faecalis*, Indocyanine Green, Therapeutic Irrigation

## Abstract

**Objectives:**

*Enterococcus faecalis (E. faecalis)*, an infecting microorganism of the root canals, is difficult to eliminate during endodontic therapy. In this study, the effect of root canal disinfection with sodium hypochlorite (NaOCl) and chlorhexidine (CHX) was evaluated on planktonic and biofilm forms of *E. faecalis* in comparison with antimicrobial photodynamic therapy (aPDT) as an alternative strategy for root canal disinfection.

**Materials and Methods:**

In this study, *E. faecalis* (ATCC 29212) was used. The experimental procedures included aPDT with curcumin (CUR) and indocyanine green (ICG) as photosensitizers, irrigation with 5.25% NaOCl, 0.2% and 2.0% CHX solutions as traditional endodontic irrigating solutions, and the control group. The antibacterial and anti-biofilm potentials were assessed by counting the colony forming units and also using the crystal violet assay, respectively.

**Results:**

According to the results, *E. faecalis* biofilm was disrupted by 65.3%, 81.0% and 92.6% using 0.2% CHX, 2.0% CHX, and 5.25% NaOCl, respectively (P<0.05). In addition, CUR- and ICG-mediated aPDT displayed a significant reduction in *E. faecalis* count (90.2% and 82.5%, respectively) and its biofilm (83.6% and 75.2%, respectively) in comparison to the control group (P<0.05).

**Conclusions:**

APDT has a high potential for elimination of *E. faecalis* and is almost equivalent to NaOCl and CHX. It can be used as an adjucnt to conventional endodontic irrigating solutions.

## INTRODUCTION

The success of endodontic therapy depends on complete elimination of existing pathogenic bacteria in the root canal system [1]. *Enterococcus faecalis* is a versatile pathogenic bacterium that plays a main role in the etiology of treated and untreated root canal infections and is highly associated with treatment failures [2].

Conventional endodontic therapy is performed via mechanical preparation of the root canal with rotary instruments, accompanied by chemical cleaning, and canal irrigation with irrigants such as chlorhexidine (CHX) and sodium hypochlorite (NaOCl) followed by the application of medicaments and sealing of root canals [3].

Endodontic irrigation solutions have antibacterial properties and can considerably decrease the numbers of bacteria. However, some of these irrigants have several disadvantages including unpleasant odor and taste, toxicity, and inability to eliminate the smear layer [4]. On the other hand, the existence of exudate, collagen, and microbiota populations decrease the effectiveness of endodontic irrigation solutions [5]. According to a recent study, the biofilms of pathogenic microorganisms are more resistant to routinely used concentrations of irrigants as compared to microbiota cells, and the efficacy of irrigants decreases by the biofilm growth [6]. Therefore, there is a need to develop a new disinfection protocol in order to to improve the results of conventional endodontic therapy.

Contrary to endodontic irrigation solutions, antimicrobial photodynamic therapy (aPDT), also known as photo-activated disinfection, photoactivated chemotherapy, photoradiation therapy, phototherapy, or photochemotherapy, is presently used as an innovative alternative for disinfection of root canals. It involves activation of a photosensitizing agent (photosensitizer) with an appropriate wavelength of light in presence of oxygen [7, 8]. Indocyanine green (ICG) is an anionic photosensitizer with an absorption peak at 810 nm, which has antibacterial effects on Gram-negative and Gram-positive oral bacteria [9]. Curcumin (CUR), another photosensitizer with an absorption peak at 430±20 nm, is a phenolic compound, which can be a suitable photosensitizer for root canal decontamination [10]. During aPDT, a photosensitizer activated via a suitable wavelength of light results in formation of reactive oxygen species such as superoxide, hydroxyl radicals, and singlet oxygen that cause the disintegration of target cells [7]. We hypothesized that CUR- and ICG-mediated aPDT would have higher efficacy for inhibition of *E. faecalis* as compared to endodontic irrigation solutions. The aim of this present study was to compare the effects of aPDT and different irrigation solutions on planktonic and biofilm forms of *Enterococcus faecalis (E. faecalis)* in vitro.

## MATERIALS AND METHODS

### Bacterial strain and growth conditions:

*E. faecalis* (ATCC 29212) was obtained from the Biological Resource Center (Tehran, Iran) and incubated in fresh brain heart infusion (BHI) broth (Merck, Darmstadt, Germany) in aerobic atmosphere at 37°C for 4–5 hours, and with an optical density (OD) of 600 nm adjusted to a concentration of 1.0×10^6^ colony forming units per milliliter (CFUs/mL), as verified by both spectrophotometry (OD_600_: 0.01–0.02) and colony counting [11].

### Endodontic irrigation solutions:

NaOCl and CHX were purchased from Sigma (St. Louis, MO, USA) and were used as the irrigation solutions. The 2.0% CHX formulation was in the form of an aqueous (water-based) solution; 0.2% CHX was prepared from 2.0% CHX by titration using sterile water.

### Preparation of photosensitizers:

Curcumin (Sigma; St. Louis, MO, USA) at a final concentration of 40 mM was prepared in 0.05% dimethyl sulfoxide. ICG (Emundo^®^, A.R.C. laser GmbH, Nurnberg, Germany) stock solution at a final concentration of 1000 μg/mL was prepared by dissolving one ICG tablet in 1.0 mL of sterile distilled water. Prior to use, the photosensitizers were filtered with a sterile syringe filter of 0.22 μm pore size and were kept under dark conditions.

### Light sources:

A light emitting diode (DY400-4; Denjoy, Jiangsu, China) at a wavelength of 450 nm with an output intensity of 1000–1400 mW/cm^2^, a diode laser (Klas DX82; Konftec, New Taipei City, Taiwan) at a wavelength of 810 nm and an output intensity of 250 mW were used in this study for CUR and ICG, respectively. The output powers were measured by a power meter (Laser Point s.r.l, Milano, Italy).

### Experiment:

**Fig. 1:**
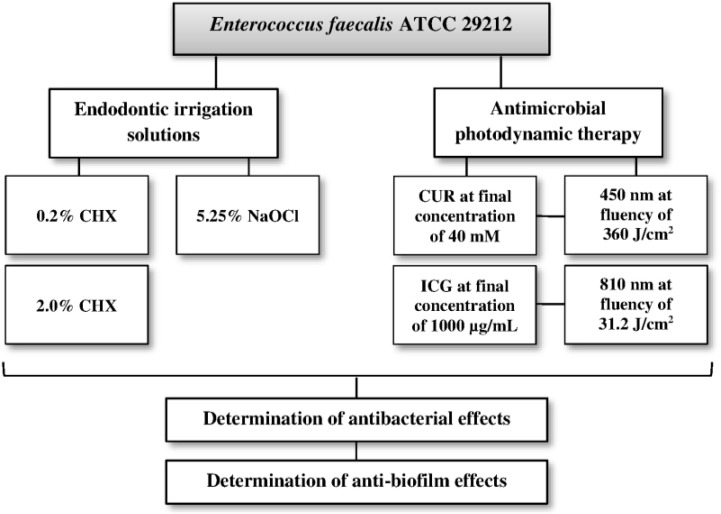
Flowchart of the testing steps in the current study

The flowchart of the experiment is shown in Figure 1. The experiments were conducted in six groups:

(1) CUR-aPDT (combined CUR and LED treatment)
(2) ICG-aPDT (combined ICG and diode laser treatment)
(3) 0.2% CHX
(4) 2.0% CHX
(5) 5.25% NaOCl
(6) Control (no exposure to either LED, diode laser, photosensitizers, or irrigation solutions)

### Antibacterial effect assessment:

The antibacterial effect against *E. faecalis* was evaluated by a broth micro-dilution method in accordance with the Clinical and Laboratory Standards Institute [11]. Briefly, the wells of a sterile 96-well round-bottomed polystyrene microplate (TPP, Trasadingen, Switzerland) were filled with 100 μL of freshly cultured *E. faecalis* (1.0 × 10^6^ CFUs/mL). According to groups, 100 μL of each photosensitizer (CUR or ICG), 100 μL of different concentrations of CHX, and 100 μL of NaOCl were added separately to designated wells. The final concentration of *E. faecalis* in each well was 1.0×10^5^ CFUs/mL. Before irradiation in groups 1 and 2, the wells were incubated in the dark for 5 minutes at room temperature (25±2°C). Groups 3–5 were not exposed to radiation, and the control group did not receive any treatment.

After incubation, the wells were irradiated using light emitting diode for 5 minutes with 360 J/cm^2^ and diode laser for 1 minute with 31.2 J/cm^2^ for CUR and ICG, respectively. Then, serial dilutions of each well in all groups were prepared in BHI broth, and 10 μL from each well containing the diluted series was spread-cultured on BHI agar (Merck, Darmstadt, Germany). The plates were incubated at 37°C for 24 h, and the CFUs/mL values of the test wells were determined using the method of Miles and Misra [12].

### Anti-biofilm effect assessment:

The biofilm forming ability of *E. faecalis* was evaluated in accordance with a previous study [13]. Volumes of 100 μL of free-floating bacteria in planktonic suspension at a final concentration of 1.5×10^8^ CFUs/mL were placed in flat-bottomed microplates (TPP; Trasadingen, Trasadingen, Switzerland), and the microplates were incubated at 37°C for 24 hours to allow biofilm formation. Anti-biofilm formation ability was evaluated according to the different treatment groups of aPDT based on photosensitizers, CHX with different concentrations, and NaOCl without irradiation as mentioned earlier. After treatment, the microplates’ contents were extracted, and the remaining suspension in the wells containing free-floating planktonic bacteria was removed by washing twice with phosphate-buffered saline (PBS; 10 mM Na2HPO4, 2 mM NaH2PO4, 2.7 mM KCl, and 137 mM NaCl at pH 7.4). The quantification of the biofilm was performed based on previous studies [13, 14]. Briefly, 200 μL of 0.1% (wt/vol) crystal violet was used to stain biofilm bacteria at room temperature for 15 minutes. After washing the wells with PBS, wells were treated with 95% ethanol for 10 minutes at room temperature and left to dry. The wells were then filled with 150 μL of 33.0% (vol/vol) acetic acid, and the absorbance was read by a microplate reader (Thermo Fisher Scientific, MA, USA) at a wavelength of 570 nm. The biofilm formation was determined according to the criteria described by a previous study [13].

### Biostatistical analysis:

All experiments were repeated at least five times, and the results were reported as mean ± standard deviation (SD).

The results of antibacterial and anti-biofilm assessments were statistically analyzed using oneway ANOVA followed by Tukey’s test. P-values <0.05 were considered statistically significant.

## RESULTS

**Fig. 2:**
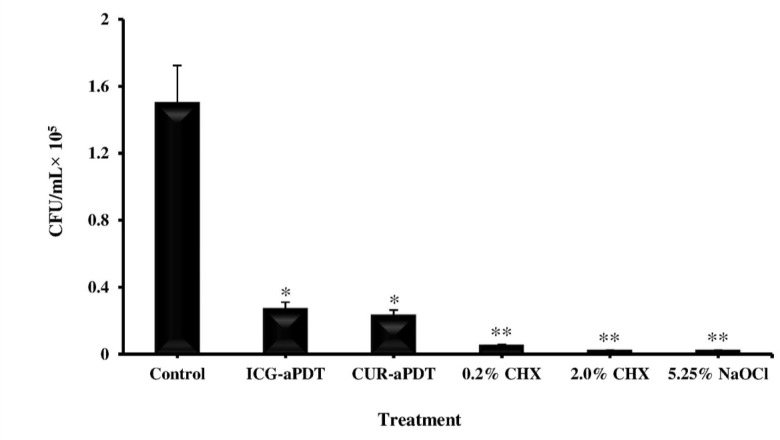
Effect of endodontic irrigation solutions and antimicrobial photodynamic therapy on *Enterococcus faecalis* colony count (Mean and standard deviation). *Significantly different from the control, P<0.05, **Significantly different from the control, P<0.001.

According to the results of our study, all treated groups displayed a significant reduction in isolated *E. faecalis* count when compared to the control groups (untreated isolates; P<0.05; Fig. 2). The minimum, maximum, mean, and standard deviation of *E. faecalis* count after different treatments are shown in Table 1. As the results showed, the *E. faecalis* count in NaOCl group was not significantly different from that in CUR- and ICG-aPDT groups, as there was no significant difference in *E. faecalis* count following the use of NaOCl and the two concentrations of CHX (P>0.05). The lowest mean value of CFUs/mL was due to the use of CHX and NaOCl solutions, followed by CUR- and ICG-aPDT groups. The highest mean value was found in the control group.

**Table 1.** Minimum, maximum, mean, and standard deviation of *Enterococcus faecalis* count in different treatment groups

	**Groups**	**Minimum**	**Maximum**	**Mean**	**Standard deviation**
**CFUs/mL**	**Control**	1.35 × 10^5^	1.65 × 10^5^	1.50 × 10^5^	± 0.12
	**0.2% CHX**	0.01 × 10^5^	0.10 × 10^5^	0.05 × 10^5^	± 0.03
	**2.0% CHX**	0.014 × 10^5^	0.027 × 10^5^	0.02 × 10^5^	± 0.004
	**5.25 % NaOCl**	0.015 × 10^5^	0.028 × 10^5^	0.02 × 10^5^	± 0.005
	**ICG-aPDT**	0.21 × 10^5^	0.37 × 10^5^	0.27 × 10^5^	± 0.06
	**CUR-aPDT**	0.15 × 10^5^	0.31 × 10^5^	0.23 × 10^5^	± 0.06
**OD 570 nm**	**Control**	3.28	3.49	3.35	± 0.10
	**0.2% CHX**	0.88	1.04	0.98	± 0.06
	**2.0% CHX**	0.60	0.78	0.69	± 0.07
	**5.25 % NaOCl**	0.41	0.66	0.52	± 0.08
	**ICG-aPDT**	1.1	1.32	1.21	± 0.07
	**CUR-aPDT**	0.92	1.09	1.01	± 0.06

All groups showed a significant reduction in the amount of biofilm produced by *E. faecalis*.

**Fig. 3:**
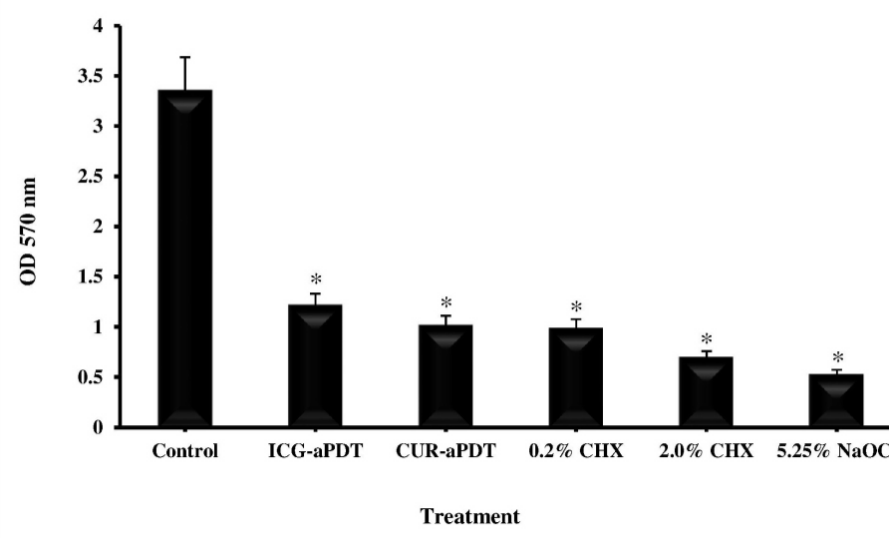
Effect of endodontic irrigation solutions and antimicrobial photodynamic therapy on *Enterococcus faecalis* biofilm. *Significantly different from the control, P<0.05.

In addition, there was no significant difference between CUR- and ICG-mediated aPDT treatment (P>0.05), and the rate of biofilm reduction in aPDT group was roughly close to the irrigation agents (P<0.05; Fig. 3). Therefore, the effects of aPDT with CUR and ICG on planktonic and biofilm forms of *E. faecalis* strains were similar close to the traditional irrigation solutions.

## DISCUSSION

There are more than 700 different microbiota in the root canal system; some of the bacterial species have not yet been identified [1]. *E. faecalis* has been the most frequently isolated species associated with endodontic infections and plays a major role in the etiology of persistent periradicular lesions after root canal treatment [2]. Disinfection of root canals with disinfecting agents is the most crucial step in elimination of microorganisms from the root canal system, dentinal tubules and the periapical region [1]. Mechanical instrumentation is the main approach for microbial reduction in the infected root canals, but achieving a root canal free from microorganisms is a difficult task. Various studies have reported that mechanical preparation with hand instruments and irrigation with saline cannot significantly eliminate the microorganisms from the infected root canals [15].

A previous study showed that 5.25% concentration of NaOCl was used in this study because there was no difference among different concentrations of NaOCl in decreasing *E. faecalis* count in the root canal system [16]. In addition, CHX was chosen because it has bacteriostatic effects at concentrations lower than 0.2% and bactericidal effects at concentrations higher than 0.2% [17].

Several studies have reported the efficacy of irrigation solutions for inhibiting the growth of microbial biofilms [17–19]. Ercan et al. [20] and Mittal et al. [21] showed that CHX was significantly more effective than NaOCl against Gram-positive bacteria. However, Bansal and Tewari [22] found that NaOCl had better antibacterial efficacy than CHX in reduction of bacterial count. In our study, the reduction of bacteria was the same in 2.0% CHX and NaOCl groups, while the level of biofilm inhibition was higher by NaOCl, which is consistent with the study by Bansal and Tewari [22], and in contrast to the results of Ercan et al. [20] and Mittal et al. [21]. Additionally, Darrag revealed that *E. faecalis* was significantly sensitive to NaOCl in planktonic form [23].

During endodontic treatment, mechanical debridement and chemical irrigation remove the majority of infecting microorganisms; however, the residual bacteria are readily detectable in the root canal because of the smear layer that reduces the effectiveness of disinfecting agents. On the other hand, the functionality of some irrigants, such as CHX, is contingent upon pH and decreases significantly in presence of organic materials [24, 25].

One of the main goals in the field of modern clinical microbiology is to develop new approaches capable of reducing the incidence of biofilm infections in the infected root canal wall. Since the efficacy of aPDT as a disinfecting agent has been previously confirmed, aPDT can be used as an alternative strategy for endodontic disinfection [7]. Regarding aPDT, it has been shown that Gram-positive bacteria are sensitive to photosensitization by many different dyes, while Gram-negative bacteria are more resistant. Moreover, aPDT not only kills the bacteria, but may also lead to detoxification of endotoxins such as lipopolysaccharide and decrease their biological activity [8].

In the present study, the two concentrations of CHX and NaOCl caused a significant reduction in bacterial viability in both planktonic and biofilm forms of *E. faecalis* more than aPDT, but it is noteworthy that there was no significant difference in viability and biofilm formation of *E. faecalis* following aPDT with endodontic irrigation solutions.

APDT as a method of disinfection cannot be applied without chemical irrigation. Based on these findings, aPDT is supposed to have an additional antimicrobial effect after root canal irrigation, especially on resistant microorganisms. Results of previous studies confirmed that the antibacterial effects of aPDT are correlated with the initial microbial count in the infected root canals, antimicrobial properties of different photosensitizers, and light irradiation time.

High concentrations of irrigation agents are highly toxic Thus, elimination of microbial biofilm prior to aPDT with adjunct use of NaOCl and CHX at a low concentration remains mandatory in order to prevent the emergence of resistant species and side effects of endodontic irrigation agents, especially in case of retreatment. Further research is needed to find appropriate aPDT settings for removal of microbiota from the infected root canals without side effects.

One limitation of this study was that only one bacterial strain was evaluated while endodontic infections are polymicrobial. In further research, aPDT should be studied as an adjunct to conventional irrigating solutions to determine if it enhances the elimination of bacteria in a shorter time.

## CONCLUSION

Following analysis of the results, the current study revealed that endodontic irrigation solutions were able to kill both planktonic and biofilm forms of *E. faecalis*. On the other hand, aPDT has a high potential for removal of *E. faecalis* and is almost equivalent to endodontic irrigation solutions although it has not yet replaced the conventional endodontic irrigation solutions.
